# Spontaneous Abdominal Wall Hematoma Treated with Percutaneous Transarterial Embolization: Diagnostic Findings, Procedural Outcome, and Efficacy—A Multicenter Study

**DOI:** 10.3390/jcm12144779

**Published:** 2023-07-19

**Authors:** Francesco Tiralongo, Francesco Giurazza, Stefano Di Pietro, Pierleone Lucatelli, Cristina Mosconi, Andrea Contegiacomo, Francesco Vacirca, Simone Ciaglia, Maria Adriana Cocozza, Davide Giuseppe Castiglione, Daniele Falsaperla, Corrado Ini’, Guido Nicola Zanghì, Antonio Granata, Massimo Venturini, Antonio Basile

**Affiliations:** 1 Radiology Unit 1, Department of Medical Surgical Sciences and Advanced Technologies “GF Ingrassia”, University Hospital Policlinico “G. Rodolico-San Marco”, University of Catania, 95123 Catania, Italy; s.dipietro@studium.unict.it (S.D.P.); f.va77@libero.it (F.V.); davidegiuseppecastiglione@gmail.com (D.G.C.); danielefalsaperla@gmail.com (D.F.); corrado.ini@gmail.com (C.I.); basile.antonello73@gmail.com (A.B.); 2 Interventional Radiology Department, Cardarelli Hospital of Naples, 80131 Naples, Italy; francescogiurazza@hotmail.it; 3 Vascular and Interventional Radiology Unit, Department of Radiological, Oncological, and Anatomo-Pathological Sciences, Sapienza University of Rome, 00161 Rome, Italy; pierleone.lucatelli@gmail.com (P.L.); simone.ciaglia@gmail.com (S.C.); 4 Department of Radiology, IRCCS, Azienda Ospedaliero-Universitaria Di Bologna, Sant’Orsola-Malpighi Hospital, 40138 Bologna, Italy; cristina.mosconi@aosp.bo.it (C.M.); mariaadriana.cocozza@studio.unibo.it (M.A.C.); 5 Department of Radiological Sciences, Università Cattolica del Sacro Cuore, Fondazione Policlinico Universitario A. Gemelli IRCCS, 00168 Rome, Italy; andrea.contegiacomo@gmail.com; 6 Department of General Surgery, University of Catania, 95123 Catania, Italy; gzanghi@unict.it; 7 Nephrology and Dialysis Unit, “Cannizzaro” Hospital, 95123 Catania, Italy; antonio.granata4@tin.it; 8 Department of Diagnostic and Interventional Radiology, Circolo Hospital, Insubria University, 21100 Varese, Italy; venturini.massimo@hsr.it

**Keywords:** embolization, radiology, interventional, hematoma, abdominal muscles, COVID-19

## Abstract

Endovascular management of abdominal wall hematomas (AWHs) is now the primary treatment option in hemodynamically stable patients, and it is often preferred to surgical interventions. The purpose of this multicentric study was to assess the safety, technical, and clinical success of percutaneous transarterial embolization (PTAE) of spontaneous AWHs to evaluate the efficacy of blind or empiric embolization compared to targeted embolization and to compare the outcome of the endovascular treatment approach in patients affected by COVID-19 and non-COVID-19 patients. We retrospectively enrolled 112 patients with spontaneous AWHs who underwent PTAE, focusing on signs of bleeding at pre-procedural CTA and DSA. Patients were separated into two groups depending on whether a blind or targeted embolization approach was used. We also divided patients into COVID-19 and non-COVID-19 groups. The mean age of the study population was 68.6 ± 15.8 years. CTA and DSA revealed signs of active bleeding in 99 and 88 patients, respectively. In 21 patients, blind embolization was performed. The overall technical success rate was 99%. Clinical success was obtained in 96 patients (86%), while 16 (14%) re-bled within 96 h. One patient reported a major peri-procedural complication. The comparison between blind and targeted embolization approaches showed no statistically significant differences in the characteristics of groups and technical and clinical success rates. No significant differences were found in the procedural outcome between COVID-19 and non-COVID-19 groups. Our study confirmed that PTAE is effective for treating spontaneous AWHs, even in COVID-19 patients. It suggests that the efficacy and safety of blind embolization are comparable to targeted embolization.

## 1. Introduction

Abdominal wall hematomas (AWHs) are characterized by the extravasation of blood inside the muscle layers of the abdominal wall [1,2,3].

AWHs may be well circumscribed by the muscle fascia or cross it and spread throughout the peritoneal or retroperitoneal space [4,5]; their development may be due to blunt or non-contusive trauma [6] or iatrogenic damage to the abdominal wall [4] or may be spontaneous.

Anticoagulant and antiplatelet therapies are the most common underlying etiologies of spontaneous AWHs. Still, spontaneous bleeding may also occur in patients with coagulation disorders, malignancies, or parietal injuries to blood vessels that can have many causes, such as hypertension, atherosclerosis, vasculitis, and hematologic disorders [3].

Spontaneous AWHs can also occur in hospitalized patients with COVID-19 [7] and represent a major cause of bleeding in these patients [8], since in this disease, a hyperfibrinolytic state, depletion of coagulation factors, and cytokine storms can be observed and can increase the risk of spontaneous bleeding [9,10].

Furthermore, thromboprophylaxis anticoagulation therapy with low-molecular-weight heparin (LMWH) in patients affected by COVID-19 can lead to hemorrhagic complications such as hematoma or bleeding [9].

Knowledge of the topographical anatomy of the abdominal wall muscles and their arterial vascularization is essential for diagnosing AWHs and their subsequent treatment [3,4,5,6].

**Figure 1:**
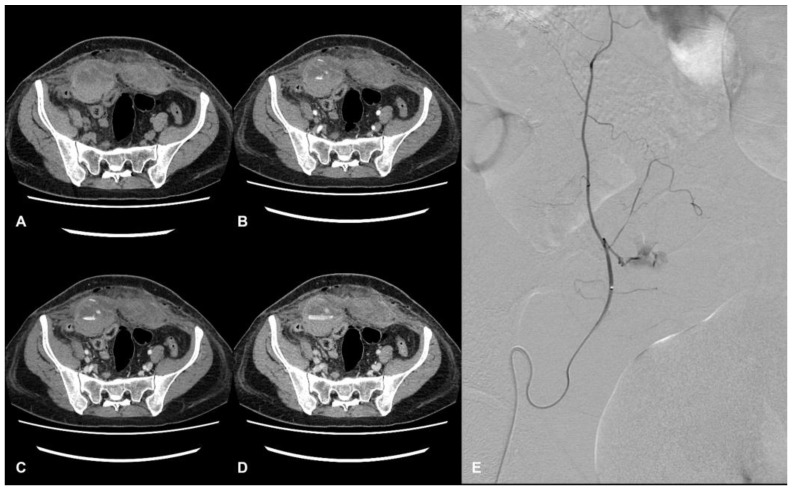
CT and DSA images of a hospitalized patient with COVID-19. Evidence of a large hematoma within rectus abdominis below the arcuate line in axial CT images. The pre-contrast phase shows the extension and location of the hematoma, showing a fluid level (hematocrit effect) (**A**). The contrast-enhanced acquisition at the arterial (**B**), portal (**C**), and delayed phase (**D**) show active bleeding within the hematoma. Angiography shows a contrast blush from branches of the right inferior epigastric artery (**E**).

Diagnosing AWHs can be challenging since signs and symptoms are often nonspecific. In this scenario, computed tomography angiography (CTA) is recognized as the gold standard for identifying and characterizing hematomas, allowing for determining the localization of the bleeding site, the extension and the dimensions of the hematoma, and the exclusion of other acute abdominal diseases (Figure 1) [7].

In COVID-19 patients, Polyaev et al. report that some specific CT signs, such as the presence of a fluid level (hematocrit effect) and the phenomenon of a “signal flare”, in the context of spontaneous hematoma, correlate with active bleeding on DSA, indicating endovascular treatment is needed [11]. The management and the treatment of AWHs can include conservative, endovascular, or surgical approaches; in hemodynamically stable patients with life-threatening AWHs, catheter-directed angiography and embolization have become the primary options for treatment [2].

Even without proof of active bleeding on digital subtraction angiography (DSA), an empiric embolization of the suspected bleeding vessel can be performed [12].

The purpose of this multicenter study was to assess the safety, technical, and clinical success of PTAE in spontaneous AWHs, to compare the efficacy of blind embolization and targeted embolization, and to compare the outcome of the endovascular treatment approach in patients affected by COVID-19 and non-COVID-19 patients.

## 2. Materials and Methods

### 2.1. Study Population and Setting

This was a retrospective, multicenter study investigating all patients with abdominal wall hematoma who underwent emergency angiographic evaluation and embolization in four Italian centers between January 2018 and November 2021.

RIS/PACS systems of the participating radiology departments were analyzed to identify all possible cases using the following keywords: embolization, transcatheter embolization, angiography, abdominal wall bleeding, abdominal wall hematoma, retroperitoneal bleeding, retroperitoneal hematoma, bleeding, and hemorrhage.

The study was conducted according to the tenets of the Declaration of Helsinki. This was a retrospective study, and ethical approval was therefore waived.

The medical records of patients identified in the search were retrieved and scrutinized retrospectively. Patients who could give consent signed a procedural consent form regarding the risks and benefits of the procedure. In cases where patients could not give consent due to the severity of the clinical condition, consent was obtained from relatives where possible.

**Figure 2:**
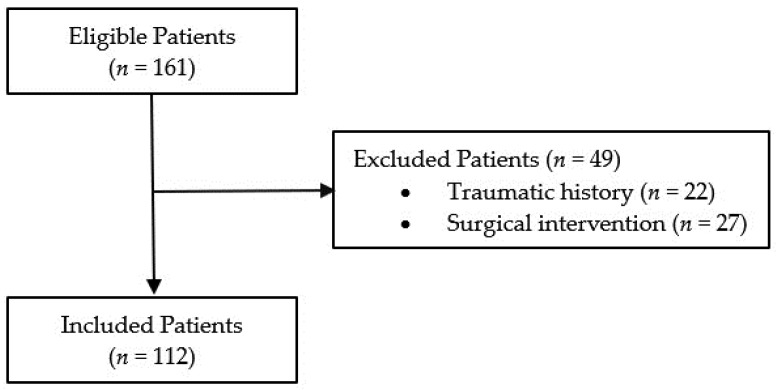
Flowchart of patient selection.

The initial search retrieved a total of 161 patients. Patients who recently underwent surgical intervention (*n* = 27) or had recent trauma (*n* = 22) within the three weeks before the diagnosis of AWHs were excluded because they were considered to have a potentially traumatic or iatrogenic hematoma. Overall, the study population included 112 patients (Figure 2).

Clinical and laboratory data such as pre-interventional hemoglobin were obtained from the digital medical records, gathered at a maximum of 12 h before the embolization procedure. All patients underwent a pre-procedural CTA before DSA. The location of the hematoma was classified as an anterior or posterior abdominal wall hematoma. Signs of active bleeding (i.e., active extravasation of contrast medium and pseudoaneurysm formation) were evaluated. A diagnostic angiography was performed in those patients without evidence of active bleeding on CTA but who presented with large hematomas and clinical and laboratory data deterioration or suspected intermittent bleeding.

DSA and TAE were performed by interventional radiologists with at least five years of experience and radiology residents attending their third or fourth year of training, with anesthesiologic support.

TAE was performed according to standard endovascular embolization techniques aiming at super-selective embolization of the bleeding vessel.

Findings considered for angiographic proof of bleeding were direct signs of active bleeding (contrast blush extravasation, focal spot of enhancement, hemorrhagic petechiae, pseudoaneurysm) or indirect signs (vessel cut-off sign or massive vasospasm).

Embolization was carried out via the microcatheter using various embolic agents: pushable or detachable coils (deployed coil diameter: 2–6 mm); temporary embolic agents such as a re-absorbable gelatin sponge (Spongostan^®^, Johnson & Johnson Medical N.V., New Brunswick, NJ, USA); a combination of coils and gelatin sponges; precipitating hydrophobic injectable liquid (PHIL 25% Terumo group); solid particles of PVA (Contour™ PVA Embolization Particles: 40–150 microns, Boston Scientific, Natick, MA, USA); micro-particles; a liquid embolic agent based on an ethylene vinyl alcohol copolymer (Menox, Meril Life, Gujarat, India); a liquid embolic agent with various viscosities and radiopacities (Squidperi, Balt international, Dusseldorf, Germany); a synthetic biodegradable cyanoacrylate basis glue (Glubran, GEM Italy, Viareggio, Italy); an ethylene vinyl alcohol copolymer dissolved in dimethyl sulfoxide (Onyx, Medtronic, Santa Rosa, CA, USA); plugs and microplugs (MVP, Medtronic^®^, Minneapolis, MN, USA).

The choice between the embolic materials was determined according to operator preference and considering the vascular anatomy and the position of the microcatheter within the target vessel.

If no signs of active bleeding were noted, blind embolization of the target vessel was performed, guided by the pre-procedural CTA findings, to decrease the arterial inflow to the hematoma and reduce the risk of recurrent hemorrhage. Other data considered for our study were the target vessel location, the procedural timing (more or less than 60 min), and the time between pre-procedural CTA and angiography.

### 2.2. Definitions

Targeted embolization is defined as “the embolization of vessels where direct or indirect signs of active bleeding are demonstrated in the DSA study” [3].

Blind embolization is defined as “the embolization of a target vessel without angiographic proof of extravasation, typically guided by CTA findings in normal-appearing vessels” [3,13].

Technical success was defined as the complete embolization of all target vessels.

Clinical success was defined as the absence of clinical, laboratory, or radiological signs of re-bleeding within a 96 h window after the procedure. Clinical failure was considered in patients who presented signs of re-bleeding (hemoglobin decrease, hypovolemic shock, or evidence of persistent bleeding at post-procedural CTA examination) and needed a new angiographic procedure followed by embolization of bleeding vessels.

Minor and major procedure-related complications were defined according to the CIRSE classification system (grade: 1 intra-procedural complication solved within the same session, no additional therapy, no post-procedure sequelae, no deviation from the ordinary post-therapeutic course; up to grade 6: death) [14].

A confirmed case of COVID-19 was defined by a positive real-time reverse-transcription PCR (RT-PCR) assay for SARS-CoV-2 on a nasopharyngeal swab.

### 2.3. Blind versus Targeted Embolization

We divided all patients into two groups depending on the embolization approach used, blind versus targeted embolization, to analyze the differences between the two study populations and to compare clinical and technical success rates and the rate of complications between these groups and these approaches.

### 2.4. COVID-19 versus Non-COVID-19 Groups

The patients were also divided into two groups—COVID-19 and non-COVID-19—depending on the presence or absence of confirmed SARS-CoV-2 infection to analyze the differences between the two study populations and to compare clinical and technical success rates and the rates of complications between these groups and these approaches.

### 2.5. Statistics

Statistical analysis was performed using the MedCalc program (MedCalc version 11.4.4.0, MedCalc Software bvba, Mariakerke, Belgium).

Continuous variables are presented as mean ± SD. Categorical variables are reported as percentages.

Characteristics of the study population were reported as mean (SD), range, and median.

For comparative statistics between the blind and targeted embolization groups’ gender, clinical success rates, and rates of complications, the chi-square test was performed.

A comparison between mean values of age, pre-procedural hemoglobin, and delay between CT and DSA was carried out using independent-samples *t*-tests. A *p*-value of >0.05 was considered non-statistically significant.

## 3. Results

A total of 161 patients were initially recruited. Patients with potentially traumatic or iatrogenic hematomas were excluded; a total of 112 patients (59 men and 53 women, mean age 68.5 ± 15.8 years) with spontaneous AWHs were included in the study (Figure 1).

The mean age of the study population was 68.5 ± 15.8 years (range 30–94 years). The mean value of pre-procedural hemoglobin in our population was 7.55 ± 3.36 g/dL. Characteristics of the study population are summarized in Table 1.

Pre-procedural CTA demonstrated signs of active bleeding within the hematoma in 99 patients (88%). In 13 patients (12%), CTA did not show signs of active bleeding.

The hematoma was located within the anterior or lateral abdominal wall (rectus abdominis, external oblique, internal oblique, and transversus abdominis muscles) in 64 patients (57%), and in the posterior abdominal wall (iliopsoas, psoas, and glutei muscles) in 48 cases (43%) (Table 1).

The mean delay between angiography and CT was 398 ± 646 min (range 15–6010 min).

The angiographic study showed direct signs of active bleeding in 88 patients (79%) and indirect signs of bleeding (cut-off vessel sign) in 3 cases (2%). In 21 patients (19%), no angiographic signs of active bleeding were found (Table 2).

PTAE was performed in one arterial territory in 82 (73%) cases, in two arterial territories in 25 (22%) cases, in three arterial territories in 3 (3%) cases, and in four arterial territories in 2 (2%) cases.

Overall, 146 arteries were embolized, corresponding to a mean of 1.3 per patient.

The embolized arteries were the inferior epigastric artery (*n* = 59), lumbar artery (*n* = 42), iliolumbar artery (*n* = 22), deep circumflex iliac artery (*n* = 12), hypogastric artery (*n* = 1), deep femoral artery (*n* = 1), intercostal artery (*n* = 3), superficial circumflex iliac artery (*n* = 2), superior epigastric artery (*n* = 1), superior gluteus artery (*n* = 2), and femoral profunda artery (*n* = 1).

**Figure 3:**
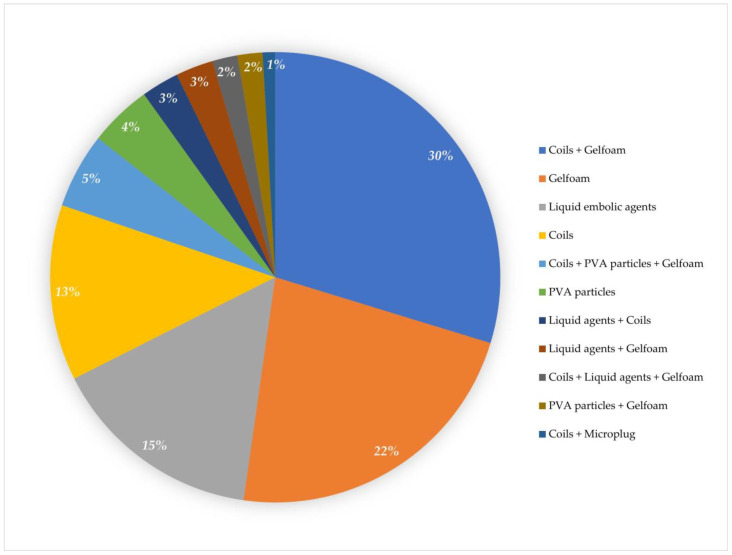
The graph shows the percentage of embolic material used for transarterial embolization.

The embolization materials used were absorbable gelatin foam in 25 (22.3%) patients, coils in 14 (12.5%) patients, a combination of gelfoam and coils in 33 (29.4%) patients, PVA particles in 5 (4.5%) patients, liquid embolic agents in 17 (15.1%) patients, a combination of coils and liquid agents in 3 (2.7%) patients, a combination of coils, gelfoam, and liquid agents in 2 (1.8%) patients, a combination of coils and microplugs in 1 (0.9%) patient, a combination of coils, PVA particles, and gelfoam in 6 (5.4%) patients, a combination of gelfoam and liquid agents in 3 (2.7%) patients, and a combination of gelfoam and PVA particles in 2 (1.8%) patients (Figure 3). 

Blind embolization was performed in 21 patients out of 112 (19%).

The mean duration of the angiographic procedure was less than 60 min in 52/105 (50%) of the procedures, while it was over 60 min in 53/105 (50%). The duration of seven procedures was not recorded nor included in our data.

In one case, diagnostic angiography demonstrated direct signs of active bleeding. TAE was attempted but was unsuccessful since it was interrupted by a major complication (dissection of the target vessel). Therefore, the technical success rate was 111/112 (99%) (Table 2).

Clinical success was achieved in 96 patients (86%) without further interventions (Table 2).

In 16 patients (14%), recurrent bleeding occurred within a 96 h time window: 12 patients had recurrent bleeding within 24 h of DSA, 2 patients re-bled after 48 h, 2 patients re-bled after 72 h, and 2 patients had a long gap between re-bleeding at 96 h.

Major complications (dissection of target vessel) were seen in only one patient. Minor complications occurred in only one case, a patient who developed a hematoma in the femoral vascular access site, as demonstrated with angiography. This was treated with manual compression at the end of the procedure. Therefore, the overall complication rate was 1.8% (Table 2).

### 3.1. Blind versus Targeted Embolization

The characteristics of the study populations of the two groups are summarized in Table 3.

The comparison between the two groups did not show statistically significant differences for gender, mean age, mean pre-procedural hemoglobin, localization of AWHs, or mean delay between CT and DSA.

In the blind embolization group, the embolic agents used were absorbable gelatin foam in 10 (47%) patients, coils in 2 (10%) patients, a combination of gelfoam and coils in 5 (23%) patients, liquid embolic agents in 2 (10%) of patients, a combination of coils and PVA particles in 1 (5%) patient, and particles alone in 1 (5%) patient. The technical success rates were 99% for the targeted embolization group and 100% for the blind embolization group.

Clinical success was obtained in 86% of patients in both groups.

The chi-squared test showed no statistically significant differences between technical and clinical success rates of AWHs treated with blind and targeted embolization (*p*-value of 0.42 and 0.72, respectively) (Table 3).

### 3.2. COVID-19 vs. Non-COVID-19 Groups

The characteristics of the two study populations are summarized in Table 4. The comparison between the two groups did not show statistically significant differences for gender, mean age, or mean delay between CT and DSA.

The independent-samples *t*-test for comparing the mean pre-procedural hemoglobin, showed a statistically significant difference between the two groups with a mean value of 6.6 ± 2.9 for the COVID-19 group and 8.55 ± 2.63 for the non-COVID-19 group (*p* = 0.02).

Signs of active bleeding on CTA were demonstrated in 88% of patients in both groups.

Moreover, DSA showed the presence of active bleeding in 13 out of 17 patients (76%) in the COVID-19 group and 78 out of 95 (82%) patients in the non-COVID-19 group, with no statistically significant differences (*p* = 0.83).

The independent-sample t-test for comparing the mean embolized arteries showed a higher mean value (1.7 ± 0.98) for the COVID-19 group than the non-COVID-19 group (1.26 ± 0.5), with a *p* = 0.006 (Table 4).

In the COVID-19 group, the embolization materials used were a combination of gelfoam and coils in nine (53%) patients, absorbable gelatin foam in two (12%) patients, coils in one (6%) patient, liquid embolic agents in two (12%) patients, and a combination of coils and PVA particles in two (12%) patients. Likewise, the duration of the procedure was less than 60 min in 29% of procedures in the COVID-19 group and 49% in the non-COVID-19 group, without statistically significant differences according to the chi-square test (*p* = 0.20).

Regarding the location, in 14 COVID-19 patients (82%), the hematoma was localized in the posterior abdominal wall compared with 34 patients (36%) in the non-COVID-19 group, and the chi-square test showed the presence of statistically significant differences (*p* = 0.0009) (Table 4).

The technical success rates were 99% for the non-COVID-19 group and 100% for the COVID-19 group without statistically significant differences (*p* = 0.32); 3 out of 17 (18%) COVID-19 patients and 13 out of 95 (14%) non-COVID-19 patients re-bled after the first PTAE. The chi-square test showed no statistically significant differences between the clinical success rates in patients with and without COVID-19 (Table 4).

## 4. Discussion

Endovascular treatment of AWHs has become the treatment of choice, especially when conservative treatment is not effective, with it being preferred to the surgical approach [2,3,4].

AWHs are more frequent in the elderly population; several studies report a mean age ranging from 70 to 71.9 years [2,4,15,16]. Hatjipetrou et al. [4] showed a mean age of 46 to 69. In our study, the mean age of 68.6 ± 15.8 years is in line with some studies found in the literature [15,16].

CTA represents the gold standard for identifying and characterizing AWHs [17,18]. In this study, CTA showed signs of active bleeding in 99 patients (88%), the same rate as that reported by Barral et al. [16] (88%); other studies reported rates ranging from 47% to 93% [2,3,12].

The location of hematomas was within the anterior or lateral abdominal wall in 64 patients (57%).

On DSA, active bleeding was demonstrated in 79% of patients; these data are consistent with the literature since some authors reported rates ranging from 70% to 85% [2,3,12,16].

In our series, the bleeding vessels most frequently identified on DSA and then embolized were the lumbar and iliolumbar arteries (42 and 22 times, respectively) for posterior AWHs and the inferior epigastric artery (59 times) for anterior AWHs; these results are in line with the results of Di Pietro et al. [3].

TAE was performed in more than one arterial territory in 30 patients, and 20 out of these 30 cases were patients with posterior AWHs. As suggested in a previous study by Di Pietro et al. [3], these data might suggest a greater complexity in treating posterior AWHs, due to the more significant number of vessels to be embolized.

Transarterial embolization can be performed using different embolic agents, which can be categorized as follows: temporary or permanent, solid (coils, plugs, microplugs), liquid (glues and polymers), used alone or in combination [19].

Nowadays, other embolizing materials, such as non-adhesive embolizing agents (NAEAs), ethylene vinyl alcohol copolymer (EVOH), polyvinyl alcohol (PVA), polylactide-co-glycolide (PGC), and hydroxyethyl methacrylate (HEMA), have gained more popularity in TAE [19,20]. In our experience, a combination of gelfoam and coils (29.4%) was the most often used material; this result is consistent with other experiences [3,7,21,22].

Becker et al., instead, reports a preference for n-butyl-2-cyanoacrylate (NBCA) as an embolizing agent, as it allows rapid and reliable occlusion of vessels, in spite of complications such as tissue ischemia, systemic or local reactions, catheter adhesion, or glue fragmentation [23].

In our study, gelfoam alone, liquid embolic agents, and coils were used in 22%, 15%, and 12.5% of cases, respectively. This heterogeneity is due to the discretion of the interventional radiologist, based on their familiarity with the embolic material and the characteristics of the bleeding vessels.

The rates of technical and clinical success reported in our experience were 99% and 86%, respectively, and these data do not differ from those reported in other studies, where technical success rates range from 96% to 100% and clinical success rates range from 65% to 93% [2,3,12,16,21,23].

Regarding blind embolization, there are a few studies in the literature concerning patients with AWHs who underwent TAE without previous demonstration of active bleeding on DSA. In agreement with our study, where blind embolization was carried out in 21 patients out of 112 (19%), Touma et al., in a systematic review, reported that 13.8% of patients with AWHs underwent empiric embolization [12], while Di Pietro et al., in a monocentric experience, reported that 21% of patients were treated with blind embolization [3].

Our results demonstrate that there was no statistically significant difference between targeted embolization and blind embolization approaches for the two study populations; the technical success rates were 99% for the targeted embolization group and 100% for the blind embolization group, and the clinical success rates were the same (86%) in both groups, without statistical significance between the two groups, agreeing with the limited literature [3].

Regarding the comparison between the COVID-19 and the non-COVID-19 group, we found a significantly lower value of mean pre-procedural hemoglobin in the COVID-19 group than the non-COVID-19 group (6.6 ± 2.9 vs. 8.55 ± 2.63; *p* = 0.02). One hypothesis explaining this difference, although further data would be necessary to support it, is that COVID-19 patients, who in most cases receive prophylactic anticoagulation with low-molecular-weight heparin, have an impaired coagulation status, and, in the case of bleeding, this could be more severe than in non-COVID-19 patients.

We report a significant prevalence of hematomas in the posterior abdominal wall in the COVID-19 group (82%). This finding agrees with Riu et al., where, in a series of spontaneous bleeding of soft tissue incidents in COVID-19 patients, 57% had bleeding in the iliopsoas and gluteus muscles [23].

The mean number of embolized arteries was 1.7 ± 0.98 in the COVID-19 group and 1.26 ± 0.5 in the non-COVID-19 group, and the comparison between the two groups showed a statistically significant difference (*p* = 0.006). This result may suggest that, in COVID-19 patients, the embolization approach could be more aggressive than in non-COVID-19 patients. This finding could explain the predominance of posterior location in the COVID-19 group since, as previously reported in the literature, posterior AWHs may be more complex to treat [3] because they are supplied by more vessels than anterior AWHs. However, this hypothesis should be considered with caution.

The comparison of technical and clinical success rates between the COVID-19 and the non-COVID-19 groups showed no statistically significant differences between the two groups. Tiralongo et al. [7], in a similar retrospective analysis performed on a smaller population, reported a technical success rate of 100% in both groups and clinical success rates of 70% for the COVID-19 group and 71% for the non-COVID-19 group.

Our results could confirm that, in agreement with Riu et al. [23], TAE is an essential and safe tool for treating spontaneous bleeding occurring in COVID-19 patients who did not benefit from conservative treatment [7,8].

Furthermore, Lucatelli et al. have recently suggested that TAE, in patients affected by COVID-19 and even without evidence of active bleeding, could be preferred to the conservative treatment [24].

We are aware that this study had several limitations, in particular the retrospective design, with a limited number of included patients (especially patients treated with blind embolization and COVID-19 patients). We could not evaluate whether patients were undergoing anticoagulant or antiplatelet treatments since these data were not available in our RIS/PACS systems or digital medical records. The most significant limitation of this study was the absence of a control group of patients with AWHs treated with conservative or surgical approaches. A further limitation of the study (because of the retrospective design) was the limited follow-up duration since patients were followed up for only 96 h after the procedure for clinical purposes.

## 5. Conclusions

Our multicenter study confirms that TAE is a safe and effective therapeutic option for the treatment of spontaneous AWHs, even in COVID-19 patients, since it has been shown to have a high technical success rate and acceptable clinical success rate. Therefore, we suggest that it be considered as a standard procedure in selected patients, even in case of the absence of angiographic evidence of active bleeding.

Moreover, our results demonstrate that the safety and efficacy of blind embolization are comparable to non-blind embolization, and we believe that the choice of performing a blind embolization can be reasonable, effective, and safe.

## Figures and Tables

**Table 1:** Characteristics of our study population and findings on CTA and DSA.

Characteristics	Value
**Age**	
*Mean ± SD*	68.6 ± 15.8 years
*Range*	30–94
**Male**	59
**Female**	53
**Pre-Procedural Hemoglobin (g/dL)**	
*Mean ± SD*	7.55 ± 3.36
*Range*	5.1–13.7
**Localization of AWH**	
*Anterior and/or lateral abdominal wall*	64 (57%)
*Posterior abdominal wall*	48 (43%)
**CT findings**	
*Active bleeding*	99 (88%)
*No proof of active bleeding*	13 (12%)
**Angiographic findings**	
*Direct signs active bleeding*	88 (79%)
*Indirect signs of bleeding*	3 (2%)
*No proof of active bleeding*	21 (19%)

**Table 2:** The outcome of PTAE.

Outcome of Percutaneous Transarterial Embolization	Value *n* (%)
**Technical success rate**	111 (99%)
**Clinical success rate**	96 (86%)
**Complications rate**	2 (1.8%)
Major	1 (0.9%)
Minor	1 (0.9%)

**Table 3:** Comparative results of blind and targeted embolization procedures.

	Targeted Embolization	Blind Embolization	*p*-Value
**Number of patients**	91	21	*-*
**Male**	48 (%)	11 (%)	0.83
**Female**	43 (%)	10 (%)	0.83
**Mean age of patients (years)**	73.36 ± 10.6	71.95 ± 16.87	0.62
**Mean pre-procedural hemoglobin (g/dL)**	8.06 ± 2.76	8.6 ± 2.8	0.96
**CTA findings**			
**Anterior hematoma**	53 (%)	11 (%)	0.80
**Posterior hematoma**	38 (%)	10 (%)	0.80
**Mean CT-DSA delay (minutes)**	413 ± 712	333 ± 217	0.62
**DSA findings**			
**Mean number of embolized arteries**	1.3 ± 0.57	1.4 ± 0.8	0.42
**Procedural timing (<60 min)**	40 (47%)	12 (60%)	0.42
**Technical success rate**	90 (99%)	21 (100%)	0.42
**Clinical success rate**	78 (86%)	18 (86%)	0.72
**Complications rate**	2 (2.2%)	0 (0%)	0.81

**Table 4:** Comparative results between non-COVID-19 and COVID-19 groups.

	COVID-19 Group	Non-COVID-19 Group	*p*-Value
**Number of patients**	17	95	*-*
**Male**	12 (71%)	47 (49%)	0.17
**Female**	5 (29%)	48 (51%)	0.17
**Mean age of patients (years)**	77.1 ± 8.8	72.3 ± 12.3	0.13
**Mean pre-procedural hemoglobin (g/dL)**	6.6 ± 2.9	8.55 ± 2.63	0.02
**CTA findings**			
**Active bleeding on CTA**	15 (88%)	84 (88%)	0.69
**Anterior hematoma**	3 (18%)	61 (64%)	0.0009
**Posterior hematoma**	14 (82%)	34 (36%)	0.0009
**Mean CT-DSA delay (minutes)**	412 ± 263	395 ± 695	0.92
**DSA findings**			
**Active bleeding on DSA**	13 (76%)	78 (82%)	0.83
**Mean number of embolized arteries**	1.7 ± 0.98	1.26 ± 0.5	0.006
**Procedural timing (<60 min)**	5 (29%)	47 (49%)	0.20
**Technical success rate**	17 (100%)	94 (99%)	0.32
**Clinical success rate**	14 (82%)	82 (86%)	0.95
**Complications rate**	0 (0%)	2 (2.1%)	0.69

## Data Availability

Not applicable.
